# Editorial: The role of nod-like receptor (NLR) family of proteins in inflammation

**DOI:** 10.3389/fimmu.2026.1793019

**Published:** 2026-02-02

**Authors:** Atika Dhar, Rishi Kumar Jaiswal, Hasan Zaki, Samithamby Jeyaseelan

**Affiliations:** 1 National Institutes of Health, Bethesda, MD, United States; 2 University of Arkansas for Medical Sciences, Little Rock, AR, United States; 3 University of Texas Southwestern Medical Center, Dallas, TX, United States; 4 Louisiana State University and A&M College, Baton Rouge, LA, United States

**Keywords:** NLRs, autoinflamatory disease, inflammasomes, inflammation, innate immunity

The immune system has been broadly divided into innate and adaptive branches, with the innate immune system serving as the initial mode of response to an infection induced by pathogen-associated molecular patterns (PAMPs) or host cell-derived molecules caused by damage-associated molecular patterns (DAMPs). The cellular molecules participating in the innate immune response may exist either as membrane bound toll-like receptors (TLRs) or intracellular Nod-Like Receptors (NLRs) to recognize either PAMPs or DAMPs. Some NLRs form multimeric protein complexes upon activation termed inflammasomes. Based on their domain structure, the members of the NLR family can be categorized into subfamilies termed as NLRA, NLRB, NLRC, NLRP and NLRX proteins (1–3). This family holds importance due to its multifaceted roles in the immune system, and a majority of the manuscripts discuss opportunities and challenges in using these molecules to augment or mitigate inflammation in multiple pathological settings.

Our knowledge on diverse functions of different NLR family proteins continues to evolve. This Research Topic makes a significant addition towards that goal. The research articles and reviews collected herein highlighted the function and significance of different NLR proteins in the context of infection and inflammation, with the NLRP3 inflammasome emerging as the central focus. Collectively, these studies expand our knowledge of the functions and biological roles of several NLRs and underscore their therapeutic potential in both human and animal diseases.

Diffuse alveolar hemorrhage (DAH) is generally associated with autoimmune conditions such as Systemic Lupus Erythematosus (SLE) and small vessel vasculitis (SVV). The underlying mechanisms contributing to DAH, characterized by leakage of red blood cells from the alveolar capillaries, remain largely unknown. In an insightful study, Andrè-Jarrot et al. investigated the role of NLRP3 inflammasome in DAH by using a well-established pristane-induced DAH mouse model. The authors demonstrated that NLRP3 inflammasome activity is important for inducing neutrophil-extracellular trap (NET) following exposure to pristane, implicating NLRP3 in lung injury. Interestingly, while NLRP3 deficiency did not protect male mice, female mice exhibited reduced severity of pristane-induced DAH, accompanied by decreased neutrophil recruitment and NET formation in the lungs. The findings reveal a female sex-specific role of NLRP3 inflammasome in DAH pathogenesis and underscore a new avenue for future investigators.

In a comprehensive review article, Liu et al. focused on the role of the NLRP3 inflammasome in atherosclerosis. Dysregulated NLRP3 inflammasome activation in arterial tissue has been previously linked to atherosclerosis development. This review article performed a systematic analysis of the existing literature on the use of Chinese herbal medicines (CHMs) in treating atherosclerosis by targeting the NLRP3 inflammasome and associated pathways. This review highlights the therapeutic benefits of the CHM, discussed their mechanism of action, and suggest the potential for safer and more targeted therapeutic strategies for using these compounds.

In another in-depth review, Sun et al. have summarized the literature concerning the role of NLRP3 inflammasome in the development of urogenital cancers, particularly bladder and renal cell carcinoma, prostate cancer, ovarian cancer, and uterine malignancies. The authors also discussed the therapeutic potential of targeting the NLRP3 inflammasome against different forms of cancer and provided an overview of specific NLRP3 inhibitors currently in preclinical or clinical development. This work serves as a valuable framework for future research exploring inflammasome-based therapies in urogenital cancers.

In addition to human diseases, inflammasomes also plays critical roles in infectious diseases affecting animals. *N. canium* is the causative agent of Neosporosis, a disease with major economic consequence for the dairy and beef industries. While cellular infection with *N.canium* has been associated with inflammasome activation and apoptosis, the pathogen-derived molecules involved in these processes remained unknown. To address this knowledge gap, Chen et. al. provided mechanistic insight into the role of the *N. canium* dense granule protein 7 (NcGRA7) in activation of the inflammasome and NF-kB pathway during infection via interaction with host cell prohibitins. This study enhances our understanding of pathogen strategies to establish infection and may guide for the development of novel therapeutic interventions.

In another extensive review, Woolls et. al. discussed the multiple regulatory roles of atypical NLR family member NLRX1 in anti-viral immunity. Their work delves on the various complex interactions between NLRX1 and relevant molecular mediators of antiviral immunity such as MAVS, FAF1 and FBXO6 and others. They comprehensively discussed various mechanisms by which NLRX1 regulates immune responses to both DNA and RNA viruses. Overall, this review provides a holistic overview of various roles of NLRX1 in anti-viral immunity and inflammation, while also pointing to the important gaps that need to be further investigated.

Finally, in their case-report, Subhi Hnaihen et. al. present a case report describing a young patient with Blau syndrome who exhibited an unusual clinical symptom of renal failure. Blau syndrome is a rare genetic disorder resulting from mutations in the *CARD15* (NOD2) gene, which is a member of the NLR family. The patients often show a characteristic triad of symptoms which is arthritis, skin rash and uveitis. The case presented by the authors, however, displayed a single symptom i.e. Arthritis and was therefore misdiagnosed as having Juvenile Idiopathic Arthritis (JIA). The authors therefore suggest a thorough assessment of family history, recognition of atypical treatment response and early genetic testing to facilitate faster diagnosis and management of Blau syndrome.

In summary, these studies presented in this Research Topic significantly expand our knowledge on the roles of NLR family proteins in infection, inflammation, and cancer. The original research articles provide mechanistic insights into how NLRs regulate inflammatory pathways and disease pathogenesis, revealing novel therapeutic targets. The review articles offer comprehensive and accessible resources that integrate current knowledge of NLR functions, signaling mechanism, interacting partners, and therapeutic strategies. Altogether, this Research Topic will enhance further interest in NLR signaling pathways and guide future research in immunology and inflammation.
